# Supplementary material for: Synergistic Action of Mild Heat and Essential Oil Treatments on Culturability and Viability of Escherichia coli ATCC 25922 Tested In Vitro and in Fruit Juice
Source: Foods. 2022 May 30;11(11):1615. doi: 10.3390/foods11111615 (PMC9180004; doi:10.3390/foods11111615)
Supplement: Supplementary file 1 [file foods-11-01615-s001.zip › foods-1725806-supplementary.pdf]

foods

Article

Synergistic Action of Mild Heat and Essential Oil Treatments on Culturability and Viability of *Escherichia coli* ATCC 25922 Tested In Vitro and in Fruit Juice

Luciana Di Gregorio [1,†], Alex Tchuenchieu [2,3,*], Valeria Poscente [1,4,†], Stefania Arioli [5], Antonella Del Fiore [1], Manuela Costanzo [1], Debora Giorgi [1], Sergio Lucretti [1] and Annamaria Bevivino [1,*]

1 Department for Sustainability, Biotechnologies and Agroindustry Division, ENEA, Italian National Agency for New Technologies, Energy and Sustainable Economic Development, Casaccia Research Center, 00123 Rome, Italy; luciana.digregorio@enea.it (L.D.G.); valeria.poscente@unitus.it (V.P.); antonella.delfiore@enea.it (A.D.F.); manuela.costanzo@enea.it (M.C.); debora.giorgi@enea.it (D.G.); sergio.lucretti@enea.it (S.L.)

2 Centre for Food and Nutrition Research, Institute of Medical Research and Medicinal Plants Studies (IMPM), Yaoundé P.O. Box 6163, Cameroon

3 Food Evolution Research Laboratory, School of Tourism and Hospitality, College of Business and Economics, University of Johannesburg, Johannesburg 2000, South Africa

4 Department of Agriculture and Forest Sciences, Università degli Studi della Tuscia, 01100 Viterbo, Italy

5 Department of Food, Environmental and Nutritional Science, Università degli Studi di Milano, 20122 Milan, Italy; stefania.arioli@unimi.it

* Correspondence: tkad01@yahoo.fr (A.T.); annamaria.bevivino@enea.it (A.B.); Tel.: +39-0630483868 (A.B.)

† These authors contributed equally to this work.

a

	Subpopulation	Growth % 1 cell/well	10 cell/well	100 cell/well
Untreated	Alive (H1-LR)	75	88	100
	Dead (H1-UL)	0	0	0
		**OEO + 55°30'**		
0 ppm	Alive (H1-LR)	62	86	90
	Dead (H1-UL)	0	12	25
50 ppm	Alive (H1-LR)	50	86	86
	Dead (H1-UL)	0	12	18
100 ppm	Alive (H1-LR)	50	58	62
	Dead (H1-UL)	0	0	12
		**OEO + 65°5'**		
0 ppm	Alive (H1-LR)	50	75	90
	Dead (H1-UL)	6	25	37
50 ppm	Alive (H1-LR)	38	50	86
	Dead (H1-UL)	0	25	25
100 ppm	Alive (H1-LR)	38	75	75
	Dead (H1-UL)	0	12	18
Negative Control	Alive (H1-LR)	0	0	0
	Dead (H1-UL)	0	0	0

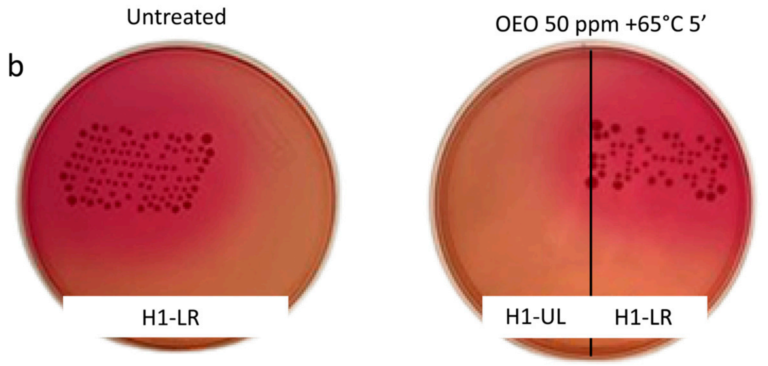

Figure S1: Flow Cell Sorting analysis of *E. coli* in different conditions and treatment. (**a**) Growth percentage expressed as cell density (OD_{600}) referred to 1 cell, 10 or 100 cells spotted into 96-well plates under treatment conditions; (**b**) Example of bacterial cells recovered from untreated and treated samples and inoculated on MacConkey agar plates using a flow sorting matrix structure of 128 cells. H1-LR: intact cells/viable cells; H1-UL: permeabilized/dead cells. *E. coli* ATCC25922 and *E. coli* ATCC25922 autoclaved at 121 °C for 15 min spotted on MacConkey Agar were used as a positive (untreated) and negative controls, respectively.
